# Comparison of three suture-bridge techniques for large or massive rotator cuff tear with delamination

**DOI:** 10.1051/sicotj/2021039

**Published:** 2021-08-16

**Authors:** Atsushi Okubo, Tadahiko Yotsumoto, Nobuyoshi Watanabe, Teruyoshi Kajikawa, Shun Nakajima, Yasushi Oshima, Norishige Iizawa, Tokifumi Majima

**Affiliations:** 1 Department of Orthopaedic Surgery, Kyoto Kujo Hospital 10 Karahashi Rajomon Minami-ku Kyoto 601-8453 Japan; 2 Department of Orthopaedic Surgery, Nippon Medical School 1-5-5 Sendagi Bunkyo-ku Tokyo 113-8602 Japan

**Keywords:** Rotator cuff tear, Delamination, Suture-bridge, En masse repair, Double-layer repair

## Abstract

*Introduction*: Rotator cuff tear with delamination is considered a risk factor for postoperative retear. The purpose of this study was to compare clinical outcomes between three repair procedures for large or massive rotator cuff tears with delamination: conventional en masse suture bridge (EMSB), double-layer suture bridge (DLSB), and the combination of DLSB with modified Debyere-Patte (DLSB + DP). *Methods*: 53 shoulders of 52 patients who had massive rotator cuff tears with delamination were categorized into three groups: EMSB (18 shoulders), DLSB (24 shoulders), and DLSB + DP (11 shoulders). The mean postoperative follow-up period was 34.6 months. Pre- and postoperative evaluations included a range of motion (ROM), Constant scores, global fatty degeneration (GFDI), and tendon integrity according to Sugaya’s classification by magnetic resonance images (MRI). *Results*: In all groups, ROM significantly improved after the procedures. Mean constant scores significantly improved: from 45.5 to 77.4 after EMSB, from 45.5 to 87.6 after DLSB, and from 46.3 to 88.0 after DLSB + DP. Significant differences were noted in postoperative Constant scores (*p* = 0.018: DLSB vs. EMSB, and *p* = 0.045: DLSB + DP vs. EMSB). The Constant pain scores were better for DLSB + DP than for EMSB (*p* = 0.012). Global fatty degeneration index (GFDI) with DLSB + DP was significantly higher than that for either EMSB or DLSB, indicating significant preoperative fatty degeneration for DLSB + DP. Retear occurred in 27.8% of the EMSB group, 12.5% of the DLSB group, and 9.1% of the DLSB + DP group. *Discussion*: Comparisons of the three groups demonstrated that DLSB and DLSB + DP achieved better clinical outcomes than EMSB for the repair of large or massive rotator cuff tears. DLSB + DP is useful for massive rotator cuff tears with severe fatty degeneration or for cases where the presence of excessive tension is anticipated when repairing the torn cuff.

## Introduction

The delamination of the rotator cuff has been described as a horizontal lesion between the superficial and deep layers [1, 2]. Delamination is observed in 36–82% of rotator cuff tear cases, and its presence is considered a risk factor for postoperative retear after rotator cuff repair [3–7]. An optimal fixation method for a rotator cuff stump with delamination has yet to be established.

Double-row double-layer fixation is reported as a repair method of the stump of delaminated rotator cuff tears. However, there is a high incidence of retear in large and massive rotator cuff tear cases [2]. The suture bridge (SB) is a tendon stump fixation method for rotator cuff tears with a strong initial fixing force and provides favorable cuff integrity with its broad coverage footprint. The en masse suture bridge (EMSB) has been used for delaminated rotator cuff tears, with an experimental study of the method demonstrating successful histological and biomechanical characteristics of cuff healing [8]. Mochizuki et al. noted the effectiveness of the double-layer suture bridge (DLSB) technique, which independently repairs the superficial layer (infraspinatus) and deep layer (articular capsule). However, they did not describe the clinical results [9]. The rotator cuff’s tear size and fatty degeneration are reported risk factors for retear after arthroscopic rotator cuff repair (ARCR) [10]. Several other repair methods of large or massive tears, such as musculotendinous advancement, have also been reported, including a modified Debeyre-Patte (DP) procedure reported effective for large or massive tears unable to be covered to the footprint by torn stumps even though sufficiently mobilized [11–13].

To our knowledge, there have been no comparative studies including muscular advancement-combined DLSB for patients with degenerative delaminated rotator cuff tears. Starting in 2010, we began using EMSB when treating delamination of the rotator cuff; however, some cases showed poor prognosis. To improve clinical results, we gradually shifted the surgical method to the double layer suture bridge method (DLSB). The purpose of our study was to retrospectively compare clinical outcomes after arthroscopic repair of delaminated rotator cuff tears which employed DLSB, EMSB, or DLSB + DP methods; and then consider an optimum repair method for the specific pathological condition.

## Materials and methods

Among patients surgically treated by arthroscopic rotator cuff repair (ARCR) at our hospital, between September 2011 and June 2018, there were 53 shoulders (52 patients) presenting massive rotator cuff tears, according to the DeOrio and Cofield classification, with delamination. These 52 patients were selected as subjects for the study. Inclusion criteria were a large or massive rotator cuff tear undergoing ARCR, and a minimum follow-up of 2 years. Exclusion criteria were patients undergoing revision rotator cuff repair, or clinical findings of instability.

The subjects were then classified into three groups based on the treatment method. The EMSB group: superficial and deep layers fixed by en bloc suturing using suture anchors. The DLSB group: superficial and deep layers individually fixed by sutures reported by Mochizuki et al. [9]. The DLSB + DP group: a modified DP procedure described [13] was added after DLSB when the rotator cuff was unable to be covered up to the footprint though sufficiently mobilized.

As shown in Table 1, the EMSB group comprised 18 shoulders of 18 patients (12 males and 6 females, mean age of 69 years (range: 58–78 years)); the DLSB group comprised 24 shoulders of 23 patients (11 males and 12 females, mean age of 69.6 years (range: 49–87 years)); and the DLSB + DP group comprised 11 shoulders of 11 patients (4 males and 7 females, at a mean age of 72.9 years (range: 67–87 years)). The mean postoperative follow-up observation durations were 31.5 months (range: 24–67 months) for the EMSB group; 34.9 months (range: 24–72 months) for the DLSB group; and 26.7 months (range: 24–36 months) for the DLSB + DP group.

**Table 1:** Patient demographics.

	EMSB (*n* = 18)	DLSB (*n* = 24)	DLSB + DP (*n* = 11)	*P*-values
Sex, *n* (males/females)	12/6	11/12	4/7	.267
Dominant/non-dominant side	12/6	19/5	7/4	.568
Age (years)	69.0 ± 6.1	69.6 ± 9.5	72.9 ± 6.1	.483
Follow-up period (months)	31.5 ± 14.0	34.9 ± 11.7	26.7 ± 4.7	.061

Notes: Data are presented as the means ± *SD*.

An institutional review board approved the study, and the enrolled subjects provided written informed consent for participation in the study.

### Surgical procedure

ARCR was performed in the beach chair position under general anesthesia on all subjects. For retention of the upper limb, an arm holder (TRIMANO Arm Holder, Arthrex, Naples, FL, USA) was used. Five portals (posterior, anterior, anterolateral, posterolateral, and upper anchor) were used for the operation. For cases of subacromial decompression, subacromial synovectomy and subacromial osteophyte resection were performed. Prior to repair, the rotator cuff was sufficiently mobilized.

**Figure 1:**
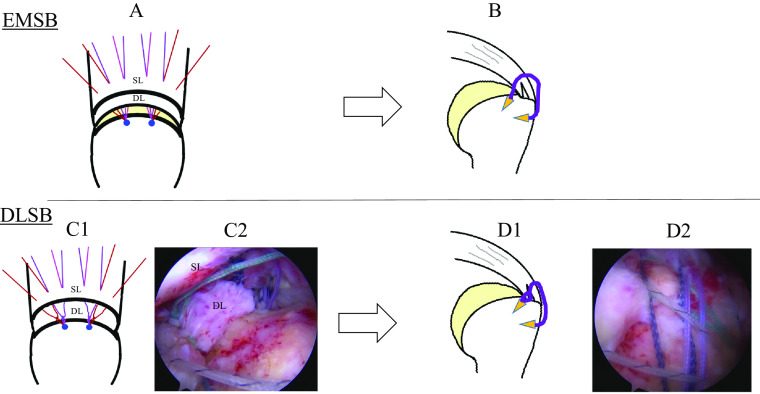
EMSB and DLSB procedures. For EMSB, a suture is passed through the superficial and deep layers (A) and bridged with lateral anchors (B). For DLSB, the superficial layer is first repaired with medial anchors (C); then, the remaining sutures are passed through the deep layer and bridged with lateral anchors (D). Abbreviations: EMSB: en masse suture bridge, DLSB: double layer suture bridge, SL: superficial layer, DL: deep layer, C2 and D2: arthroscopic images of the right shoulder as viewed from the posterolateral portal.

**Figure 2:**
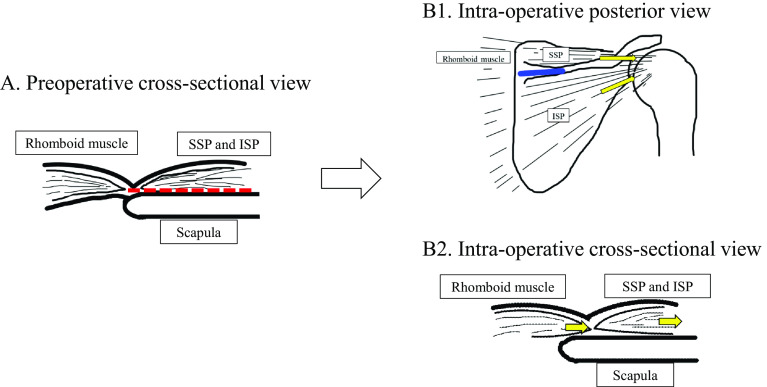
Schema of modified Debyere-Patte (DP) technique. (A) Preoperative cross-sectional view. (B1) Intra-operative posterior view of the right shoulder; (B2) Intra-operative cross-sectional view. Red dotted line: SSP and ISP, while maintaining continuity with the rhomboid fascia, were detached from the medial edge of scapular and advanced laterally. Blue line: skin incision. Yellow arrow: pulling direction of torn tendons with the rhomboids. Abbreviations: SSP: supraspinatus muscle, ISP: infraspinatus muscle. (Modified illustration of Morihara et al. [13]).

For the rotator cuff repair method, 1–2 medial anchors and 2–3 lateral anchors were set corresponding to the tear size using suture anchors (HEALIX, DePuy Synethes Mitek Sports Medicine, Raynham, MA, USA). For EMSB, the superficial and deep layers were fixed en bloc using a medial anchor thread (Figure 1). For DLSB, first, the medial anchor thread was passed through the deep layer to fix and suture by adding sliding knots and two half-hitches, as described by Mochizuki et al. [9]; then, all threads were passed through the superficial layer without cutting the anchor thread and fixed using the lateral anchor (Figure 1). In cases unable to be covered up to the footprint despite the rotator cuff being sufficiently mobilized, an approximately 3 cm transverse skin incision was made outward from the medial border of the spine of the scapula following the method reported by Morihara et al. [13]. This modified DP procedure dissecting the supraspinatus and infraspinatus muscles was done while maintaining continuity to the fascia of the rhomboideus muscle (Figure 2). Then, suture fixation of the stump was performed by DLSB.

### Rehabilitation

In the EMSB and DLSB groups, a postoperative shoulder abduction brace was attached for 3–4 weeks, corresponding to the tear size. In the DLSB + DP group, the abduction brace was attached for 6 weeks following surgery. Passive range of motion (ROM) training was initiated on the day after surgery, and active ROM training was initiated upon removal of the abduction brace. Resistance exercise was initiated 12 weeks after surgery.

### Clinical outcomes

Clinical outcome assessments included pre- and postoperative ROM (flexion, abduction, external rotation, internal rotation), Constant scores, and the global fatty degeneration index (GFDI). The latter, representing the preoperative degree of fatty infiltration into the rotator cuff as observed on MRI, was defined as the mean for the supraspinatus, infraspinatus, and subscapularis muscles, according to the Goutallier classification [14, 15]. Postoperative cuff integrity was evaluated using Sugaya’s classification, with Types IV and V regarded as retear [16].

### Statistical analysis

Statistical analysis was done using EZR (Ver. 1.50, Saitama Medical Center, Jichii Medical University, Saitama, Japan). For all three groups, pre- and postoperative ROM and Constant scores were analyzed using the Mann-Whitney *U*-test; Kruskal-Wallis was used for pre- and postoperative comparisons, and Fisher’s test was used for comparison of the incidence of retear, and a *p*-value of < 0.05 was considered statistically significant.

## Results

### Range of motion

Each surgical group showed significant postoperative active ROM improvement, including flexion, external rotation, and internal rotation degrees (Table 2). In the EMSB group, mean ROM before the operation was flexion (Flex) 105.6°, external rotation (ER) 49.7°, internal rotation (IR) L 4.8; after the last follow-up, it was Flex 158.9°, ER 67.2°, and IR L 2, respectively. In the DLSB group, ROM improved from Flex 109°, ER 47.2°, IR L 4.5 to Flex 159.6°, ER 71.4°, and IR L 2, respectively. In the DLSB + DP group, ROM improved from Flex 108.2°, ER 43.2°, IR L 3.6 to Flex 149.1°, ER 69.1°, and IR L 1.3, respectively. No significant differences were noted in either pre- or postoperative ROM improvement among the three groups (Table 2).

**Table 2:** Active range of motion before and after treatment.

	EMSB	DLSB	DLSB + DP	*P*-values
Flex				
Pre-operation	105.6 ± 50.0	109 ± 30.1	108.2 ± 43.2	.979
Last follow-up	158.9 ± 20.5	159.6 ± 19.7	149.1 ± 26.4	.509
ER				
Pre-operation	49.7 ± 16.8	47.2 ± 20.0	43.2 ± 25.6	.623
Last follow-up	67.2 ± 13.7	71.4 ± 14.9	69.1 ± 10.8	.4
IR				
Pre-operation	L 4.8	L 4.5	L 3.6	.27
Last follow-up	L 2	L 2	L 1.3	.502

Note: Data are presented as the means ± *SD*. Abbreviations: Flex: degree of flexion; ER: degree of external rotation; IR: internal rotation; L: Lumbar spine.

### Constant scores

**Figure 3:**
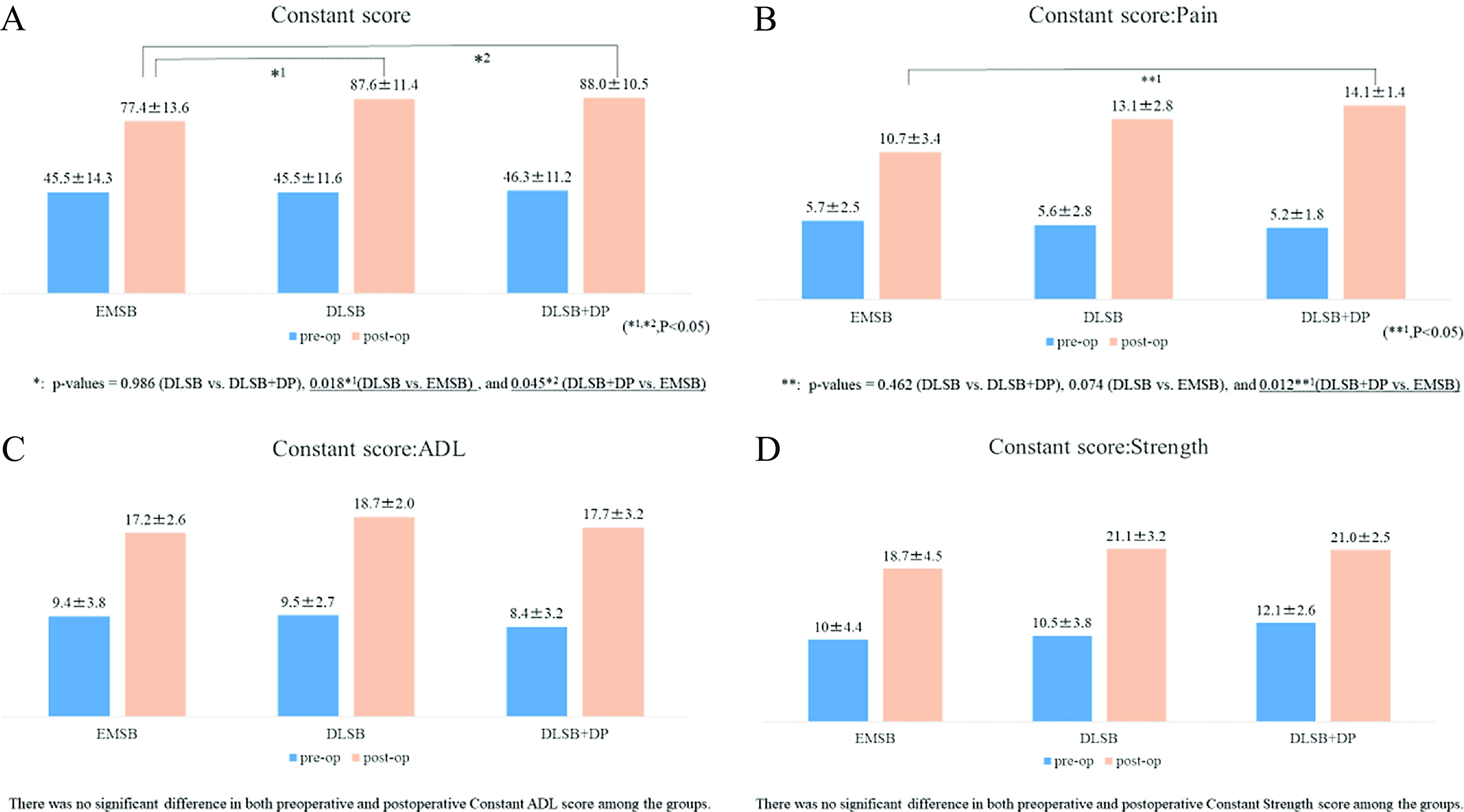
Clinical outcome assessment: Constant Scores. (A) Significant improvements were noted in the postoperative Constant scores as between the EMSB and DLSB groups (*p* = 0.018), and between the EMSB and DLSB + DP groups (*p* = 0.045). (B) Postoperative pain Constant score: a significant difference was noted in the postoperative pain between the EMSB and DLSB + DP groups (*p* = 0.012). (C) Postoperative ADL. (D) Strength of the constant scores. No significant differences were noted in the postoperative ADL and strength of the Constant scores among the three groups.

The mean Constant scores for the EMSB, DLSB, and DLSB + DP groups, respectively, were 45.5 ± 14.3, 45.5 ± 11.6, and 46.3 ± 11.2 before surgery, and 77.4 ± 13.6, 87.6 ± 11.4, and 88.0 ± 10.5 after surgery achieving favorable improvement in all groups (Figure 3A). Significant differences were noted in postoperative Constant scores between the EMSB and DLSB groups (*p* = 0.018) and between the EMSB and DLSB + DP groups (*p* = 0.045). In addition, a significant difference was noted in the postoperative pain of the Constant score between the EMSB and DLSB + DP groups (*p* = 0.012) (Figure 3B). No significant differences were noted in postoperative ADL and strength of the constant scores among the three groups (Figures 3C and 3D).

### Global fatty degeneration index

The mean preoperative GFDI was 1.52 ± 0.4, 1.80 ± 0.5, and 2.28 ± 0.4, respectively, for the EMSB, DLSB, and DLSB + DP groups, with the latter group significantly higher than the other two, and indicating significant preoperative fatty degeneration (*p* = 0.015: DLSB vs. DLSB + DP, and <0.001: DLSB + DP vs. EMSB) (Table 3).

**Table 3:** Radiological findings on MRI.

	EMSB (*n* = 18)	DLSB (*n* = 24)	DLSB + DP (*n* = 11)	*P*-values
Preoperative GFDI	1.52 ± 0.4^1^	1.80 ± 0.5^2^	2.28 ± 0.4^1^,^2^	≤.001*
Postoperative retear, *n* (%)	5 (27.8%)	3 (12.5%)	1 (9.1%)	.395

Notes: Data are presented as the means ± *SD*. Abbreviation: GFDI: global fatty degeneration index.

* *p* < 0.05.

1 *p =* 0.015 (DLSB vs. DLSB + DP), 0.107 (DLSB vs. EMSB),

2 *p* < 0.001 (DLSB + DP vs. EMSB). GFDI in the DLSB + DP group was significantly higher than that of both EMSB and DLSB groups.

### Postoperative cuff integrity

Postoperative retear occurred in 5 (27.8%), 4 (16.0%), and 1 subject (9.09%), respectively, of the EMSB, DLSB, and DLSB + DP groups, reflecting the low incidence of retear in the DLSB + DP group. No statistically significant differences among the groups were found (Table 3), and no other postoperative complications were noted.

## Discussion

Large and massive rotator cuff tears with delamination are problematic due to the risk of retear because of the anatomical characteristics of the horizontal superficial and deep layers [1–7]. Various surgical repair procedures have been used though an optimal surgical procedure has yet to be determined [2, 8, 9]. In this study, three surgical procedures, EMSB, DLSB, and DLSB + DP, were compared and investigated for their clinical outcomes treating massive rotator cuff tear. The retear rate was the lowest for the DLSB + DP group, even though its mean preoperative GFDI was the highest.

There were limitations to our study in that it was a retrospective investigation, there was a small sample size, and a lack of a priori sample size calculation. In addition, the timing for the surgical choices of EMSB and DLSB may have influenced clinical outcomes. However, to our knowledge, no reported studies are comparing the EMSB and DLSB methods with the muscular advancement-combined DLSB method for patients with massive degenerative delaminated rotator cuff tears.

The suture bridge method reportedly provides excellent rigidity compared with the single-row or double-row methods because of greater contact area and contact pressure [2, 17–19] and has been frequently used for rotator cuff tear. However, the incidence of reported retear remains high in cases of massive rotator cuff tear with severe fatty degeneration of the rotator cuff [15], and an optimum treatment method remains controversial though several treatment methods were reported for irreparable rotator cuff tear by primary repairs, such as partial repair, patch, musculotendon transfer, muscular advancement, and superior capsular reconstruction.

Delamination is often observed, which tends to show tearing in the posterior region [20]. From a histological standpoint, there has been no consistent agreement on the developmental mechanism of the two-layered delamination structure [5]. The rotator cuff is comprised of five histologically different layers, and delamination tends to develop between the 2nd and 3rd layers with different fiber distributions [21, 22]. Delamination is a risk factor for postoperative retear after rotator cuff repair, thus special attention to its presence and extent is considered important [3–5]. Delamination repair methods have included suturing the superficial and deep layers en bloc [23], individually suturing superficial and deep layers, and suturing only the superficial layer [9, 24, 25]. Mochizuki et al. reported that the superficial and deep layers should be individually sutured as their footprints are anatomically different [9]. A study describing the usefulness of DLSB, as compared to EMSB, concluded the advantage came from repairing the superficial and deep layers to an anatomically appropriate footprint [25]. Cha et al. stated risk of retear in cases with severe rotator cuff degeneration even when the dual-layer method was used, suggesting the need for an alternative suture method and additional treatment for severe degeneration of the rotator cuff [24].

For the subjects of this study, DLSB was done in combination with the modified DP method for cases considered irreparable by the primary treatment. No significant differences were noted in postoperative ROM among the EMSB, DLSB, and DLSB + DP groups. Still, the postoperative Constant scores for the EMSB group were significantly inferior to those for the DLSB and DLSB + DP groups. The outcome of pain of the Constant scores in the EMSB group, which had the highest incidence of retear, was significantly inferior to that in the DLSB + DP group, which had the lowest incidence of retear. Statistically, the incidence of retear was not significantly different between the three groups. However, in the DLSB + DP group, the retear ratio was the lowest and the preoperative GFDI the highest and significantly higher than that for DLSB. This suggests the efficacy of the modified DP method in reducing the tension of the repaired rotator cuff and possibly of DLSB + DP preventing retear in cases with severe degeneration of the rotator cuff. To further verify our findings, a long-term outcome evaluation is considered necessary.

## Conclusion

DLSB method is considered a better procedure for the successful anatomical repair of a delaminated rotator cuff tear of the shoulder. Further, the DLSB + DP method may be a useful surgical procedure for patients with a large-sized tear, severe fatty degeneration of the rotator cuff, or when excessive tension is present when repairing the rotator cuff.

## Conflict of interest

The authors declare that they have no conflicts of interest in relation to this article.

## Funding

The authors received no special funding related to this study.

## Ethical approval

The institutional review board of Kujo Hospital, Japan, approved this study.

## Informed consent

All subjects were provided explanations and signed a written consent form regarding this study.

## Authors’ contributions

A. Okubo: Designed the study, performed operations, acquired data, and analyzed results. T. Yotsumoto: Provided clinical advice and critically revised the contents of the manuscript. N. Watanabe, T. Kajikawa, and Shun Nakajima: Participated in the clinical treatment of patients and the improvement of this manuscript.Y. Oshima and N. Iizawa: Made critical comments on the contents of the manuscript and assisted in its completion. T. Majima: Comprehensively reviewed and gave suggestions to improve this manuscript.
